# Comparing Vector-Borne Disease Surveillance and Response in Beijing and the Netherlands

**DOI:** 10.5334/aogh.3672

**Published:** 2022-07-26

**Authors:** Charlotte Onstwedder, Jerome Lock-Wah-Hoon, Sigrid van Dorp, Marieta Braks, Liselotte van Asten, Yang Zheng, Thomas Krafft, Ying Tong, Wim van der Hoek, Qi-Yong Liu, Eva Pilot, Quanyi Wang, Ewout Fanoy

**Affiliations:** 1 Department of Health, Ethics & Society, Care and Public Health Research Institute CAPHRI, Faculty of Health, Medicine and Life Sciences, Maastricht University, Maastricht, The Netherlands; 2 Centre for Infectious Diseases, Epidemiology and Surveillance, National Institute for Public Health and the Environment, Bilthoven, The Netherlands; 3 Center for Zoonoses and Environmental Microbiology, National Institute for Public Health and the Environment, Bilthoven, Netherlands; 4 Institute for Infectious Disease and Endemic Disease Control, Beijing Center for Disease Prevention and Control, Beijing, China; 5 Institute of Disinfection and Vector Control, Beijing Center for Disease Prevention and Control, Beijing, China; 6 State Key Laboratory of Infectious Disease Prevention and Control, Collaborative Innovation Centre for Diagnosis and Treatment of Infectious Diseases, National Institute for Communicable Disease Control and Prevention, Chinese Centre for Disease Control and Prevention, Beijing 102206, China; 7 Public Health Service, region Rotterdam-Rijnmond, Rotterdam, The Netherlands

**Keywords:** vector-borne diseases, preparedness, surveillance, response, Beijing, the Netherlands, IHR

## Abstract

**Background:**

Climate change, environmental change, and globalization affect the geographical distribution of vector-borne diseases. Temperate regions should be prepared for emerging diseases and learn from each other’s experiences.

**Objective(s):**

The vector-borne disease preparedness in two regions, Beijing and the Netherlands, were compared in order understand their similarities and differences leading to learning points on this complex topic.

**Methods:**

A comparative study was performed using interviews with vector-borne disease experts from Beijing and the Netherlands and supplemented by literature.

**Findings:**

In Beijing, syndromic surveillance is a priority for the identification of suspected vector-borne disease cases. In the Netherlands, the main surveillance emphasis is on laboratory confirmed vector-borne disease cases. Vector-surveillance at potential points of entry and other high-risk locations is performed according to the International Health Regulation (2005) in both settings. Beijing controls invasive and native mosquitos, which is not the case in the Netherlands. In Beijing, vector surveillance is performed to measure mosquito density around hospitals, this is not observed in the Dutch setting. Health risks posed by ticks are a priority in urban areas in the Netherlands, and the public is educated in self-protection. In contrast, ticks seem to occur less often in Beijing’s urban areas.

**Conclusions:**

The vector-borne disease context framework allowed us to compare the vector-borne disease preparedness between Beijing and the Netherlands, despite differences in vector-borne disease challenges. We can learn valuable lessons concerning surveillance and early detection of emerging vector-borne diseases when comparing the preparedness between different regions.

## Background

Vector-borne diseases globally cause more than 700 000 yearly deaths and are responsible for 17% of all deaths from infectious diseases. They are transmitted to humans by vectors such as mosquitos and ticks [1]. The interactions between vectors (diversity, density), hosts (humans, animal reservoirs), pathogens (viruses, bacteria, protozoa), and biotic and abiotic components of the environment (climate, geographical location) are intricate [2]. Climate change, environmental change, and globalisation increase the risk of the introduction of vectors and vector-borne diseases to novel regions, as reflected by recent malaria, Zika and yellow fever outbreaks in China and dengue fever clusters and chikungunya outbreaks in Southern Europe [3,4].

Surveillance is defined as the “continuous, systematic collection, analysis and interpretation of health-related data needed for planning, implementation and evaluation of public health practice” [5]. A distinction is made between surveillance based on diagnosed notifiable disease cases, which are confirmed with laboratory tests (such as serologic assays, molecular techniques and microscopy), and syndromic-surveillance which is based on non-specific health indicators (e.g. respiratory symptoms not further defined, or sales figures of over the counter medication). Syndromic surveillance provides timely insight into current community health status, facilitating early warning and response [5,6,7]. Accurate and timely surveillance of vectors, pathogens, and their associated diseases is required to inform control measures [8].

The International Health Regulations (IHR) focus on a global surveillance and response system for infectious diseases to strengthen prevention and response in public health emergencies of international concern, such as geographical shift in vector-borne diseases. The IHR suggest improvements in surveillance and response systems by refining vector-borne disease control measures, and utilising approaches such as syndromic surveillance to improve early warning and outbreak response capacities [9]. Besides IHR suggestions, also collaboration between geographic regions are crucial in achieving global health security [10]. One such partnership was formed between the Ministry of Health of the People’s Republic of China and the Dutch Ministry of Health, Welfare and Sport. This Memorandum of Understanding allowed us to share experiences and find opportunities for development. With this in mind we investigated the similarities and differences between the vector-borne disease surveillance and response systems in Beijing and the Netherlands.

Being more similar in (population) size and climate, comparing the Netherlands with Beijing is better feasible than comparing it with the whole of China. Beijing is a municipality consisting of 16 smaller districts within the People’s Republic of China (population size ~21.893.095; of which 65% residents in the urban core). It covers an area of ~16.500 km^2^ and has many mountains and several larger waterways. The continental Netherlands is a country in Western Europe, subdivided in 12 provinces and covers an area of ~41.500 km^2^ (population size ~17.442.000; of which 92% residents in the urban core). It lays partly below sea level and is mainly flat. Both Beijing and the Netherlands have a temperate climate with continental influences, however, Beijing is known for its monsoons in summer and the Netherlands is prone to winter storms due to maritime influences [11,12,13].

Both regions have multi-layered infectious disease surveillance systems (Supplement 1), with national coordination and supervision in Beijing by the National Health and Family Planning Commission (NHFPC) and in the Netherlands by the Dutch Ministry of Health, Welfare and Sport. In the Netherlands data is analysed by the Dutch National Institute for Public Health and the Environment and by regional public health services. Beijing has 3 levels for data analysis at CDCs namely also at prefecture and county level [8].

We acknowledge the fact that these regions have incomparable disease risks, climates and land usage. However, we aim to compare their response- and surveillance systems using a tool that assesses the preparedness for vector-borne diseases in distinct regions [14].

## Methods

A comparative study was conducted to investigate vector-borne disease experience and preparedness in Beijing and the Netherlands. Interviews with public health experts in each region were conducted and compared. A narrative literature review was performed to find complementary information.

### Sample population

One-to-one interviews were conducted with public health experts in vector or vector-borne disease surveillance and/or response in the first half of 2018 in Beijing and the Netherlands. The experts were sampled using a purposive sampling technique combining convenience sampling with suggestions from key informants. In total 19 experts from interdisciplinary fields (e.g. biologists, epidemiologists) were included, from which 10 were employed in the municipality of Beijing and 9 in the Netherlands (Supplement 2). Additionally, 2 conferences with experts from the Dutch Institute for Public Health and the Environment (RIVM) and China- and Beijing CDC were held in 2018 and 2019.

### Data collection

The interviews were primarily conducted in Dutch and English, with translator present at the interviews in Beijing if the experts preferred answering in Chinese-Mandarin. All interviews were based on an interview guide constructed around a vector-borne disease surveillance feedback framework developed by Braks et al. [14], forming 4 dimensions for comparison:

1. Monitoring and surveillance
2. Research
3. Harmonisation and prioritisation
4. Response and action

These dimensions were identified as helpful comparing vector-borne disease surveillance and response by project team members from both Beijing and the Netherlands.

Additionally, scientific databases (e.g. PubMed, Google Scholar) and official documents (e.g. EU policy documents, WHO articles, CDC Beijing publications, RIVM manuscripts) were searched with key terms (Supplement 3) to obtain information relating to vector-borne diseases, tick- and mosquito-borne diseases, early prevention and vector-borne disease surveillance systems in the Netherlands and Beijing (China). Collected articles were used for complementary information.

### Data analysis

The audio-recorded expert interviews were transcribed using Google Docs speech recognition and ATLAS.ti version 1.6.0 (qualitative analysis software) to analyse the thematic content. All interviews were anonymised before transcription. In this comparative study, the interviews were reviewed and organised according to the four dimensions developed by Braks et al. [14]. A comparative analysis of the transcripts was carried out and complemented with literature. During the conferences preliminary findings were presented and discussed, this was conducted to check the correctness of given statements and to add nuances where needed.

## Findings

### Vector-borne disease contexts

It is relevant to prepare for potential future vector-borne disease threats. To prepare effectively, prioritisation of threats is imminent. This prioritising of threats can be performed systematically using a developed context matrix [14]. We recognise 5 contexts using the context matrix (Supplement 4) based on different combinations of 3 elements in a given geographical area: 1) autochthonous human disease case(s) presence, 2) infectious pathogen presence (either imported or locally present in human/animal cases and/or vectors) and 3) presence of an established vector population (Table 1). In the absence of 1 or more of the 3 elements, the threat level decreases [14]. A determined context always reflects the current status of knowledge. Diseases, vectors or pathogens can shift from one context to another if more knowledge becomes available or when they are introduced in the given geographical area (if this is measured or noticed in surveillance).

**Table 1:** Vector-borne disease contexts based on the current presence (√) or absence (–) of endemic (human) disease, pathogen and vector (Braks et al., 2011) with (non-exhaustive) disease examples.


CONTEXT	AUTOCHTHONOUS DISEASE CASE(S)	PATHOGEN (AUTOCHTHONOUS/IMPORTED)	VECTOR (ESTABLISHED)	EXAMPLES BEIJING	EXAMPLES THE NETHERLANDS

**1**	**√**	**√**	**√**	Lyme disease, Japanese encephalitis	Lyme disease, tick-borne encephalitis

**2**	**–**	**√**	**√**	*vivax*-malaria, tick-borne encephalitis	*vivax*-malaria

**3**	**–**	**–**	**√**	West Nile fever, chikungunya, dengue	Rift Valley fever

**4**	**–**	**√**	**–**	yellow fever, Zika	Yellow fever, dengue, chikungunya, Zika

**5**	**–**	**–**	**–**	Crimean Congo haemorrhagic fever	Japanese encephalitis, Crimean Congo haemorrhagic fever


This framework allows us to compare the regions’ preparedness, despite differences in the diseases, pathogens, and vectors present. We investigated the preparedness for context 1 to context 5 vector-borne diseases, using selected tick- (Lyme disease, tick-borne encephalitis and Crimean Congo haemorrhagic fever) and mosquito-borne diseases (Zika, chikungunya, dengue, malaria, Japanese encephalitis, yellow fever and West Nile fever) as examples in Beijing and the Netherlands.

### Monitoring and surveillance

Both regions perform vector and human case surveillance. This is differentiated between research activities and the routine public health monitoring and vector monitoring for surveillance and response.

#### Vector-surveillance

In both regions, vector monitoring is described as an easily adaptable process. It is scaled up when there is a risk for increased vector activity, such as favorable environmental and climate conditions. Activities such as tick-collection are usually project-based instead of embedded into routine vector-surveillance activities.

In the Netherlands, citizen science projects provide vector survey information. Based on the information-for-data principle, such projects involve a reciprocal relationship between research institutions and citizens. Examples are the Nature Calendar Study, in which citizens monitored ticks in 20 locations monthly over 15 years, and “Tick radar,” a web-based educational tool where citizens report tick bites [15]. In return for such data, citizens receive information on areas with high reported tick bites. In contrast, no citizen science projects were identified in Beijing [16].

In both regions, mosquito surveillance is performed to prevent establishment and transmission at points of entry such as ports and airports. The threshold for initiating a response to invasive mosquitoes is set at “zero mosquitoes” by the IHR. Accordingly, surveillance is implemented in both regions to maintain zero status within a 400 meter range of sampling sites [17]. If vectors are found within this range, public health authorities must be notified and the situation controlled.

#### Human disease

Human disease surveillance is coordinated at national and regional levels in both regions, but Beijing has an additional lower district level while the Netherlands has an additional international European level. Beijing reports notifiable vector-borne diseases to the highest level of monitoring and control, the national China CDC (covering 34 provinces). In contrast, RIVM reports to the European Centre for Disease Prevention and Control (ECDC, covering 31 countries). Both regions have nationally determined notifiable disease lists, but at the European level, surveillance practices are not uniform, reflecting differences in reported data between the EU/EEA Member States [8].

In Beijing, surveillance for human cases occurs via passive syndromic surveillance, laboratory confirmed surveillance and active surveillance during outbreaks. Passive syndromic surveillance takes place in hospital clinics specialised in vector-borne diseases. Commercial laboratories offer diagnostic services for vector-borne diseases; however, results need to be confirmed by the Beijing CDC laboratory.

Laboratory confirmation is the cornerstone for vector-borne disease surveillance in the Netherlands. All permanent residents are registered at a general practice. A general practitioner (GP) authorises access to hospital care and specialist care referrals.

A selection of vector-borne diseases in the two regions is discussed below, using the context framework.

**Context 1.** Endemic human disease cases, with both the vector and pathogen present

Lyme borreliosis is endemic in both regions where autochthonous transmission is indicated in rural Beijing (tick: *Ixodes persculcatus)* and both in rural and urban Netherlands (tick: *Ixodes ricinus)*, positioning itself in context 1 (Table 1) [18,19]. A research driven survey for academic purposes on Lyme borreliosis found a high rate of positive antibody tests (12% +/– 2%, N = 1 000) in rural clinics of Beijing. While observed infection rates of Lyme borreliosis in rural Beijing keep increasing, academic surveys indicated an absence of risk for the urban core [18]. In the Netherlands stabilisation of early Lyme borreliosis (~158 infections per 100 000 infections annually) was reported in 2014 after 15 years of continuous increase [20]. In 2012, a citizen science survey indicated that 1 in 5 tick bites were reported to originate from urban areas, such as in communal parks and playing fields [21]. Besides Lyme disease, incidental autochthonous human cases of tick-borne encephalitis, PCR-positive ticks and serology-positive animal reservoirs of the causing virus have been reported in the Netherlands since 2015 [22].

Japanese encephalitis, a zoonotic mosquito-borne disease placed in Context 1 for the Beijing setting, is transmitted by *Culex tritaeniorhynchus* in rural and suburban areas due to its contact with animal reservoirs such as pigs and water birds. Intensified transmission of Japanese encephalitis virus in the animal reservoir occurs during the rainy season with the main mechanism driving mosquito densities attributed to the flooding of irrigation systems in rice cultivation areas [23]. Since 2014, autochthonous human Japanese encephalitis cases have declined substantially due to compulsory vaccinations in the general population [24].

For the last half-century until 2018, no autochthonous human cases of mosquito-borne diseases have occurred in the Netherlands. However, Usutu virus has been circulating in bird populations since 2016 [25].

**Context 2.** Vector and pathogen present, but no autochthonous human disease cases

Imported cases might pose an outbreak risk when they introduce a pathogen to competent vectors that are already established (Context 2). In both regions malaria is an example, with females of the *Anopheles* genus transmitting *Plasmodium vivax*, the causal parasite of *vivax*-malaria [26].

In Beijing, the *Anopheles hyrcanus* is a widespread competent vector of *Plasmodium vivax* [27]. However, no autochthonous cases of *vivax*-malaria have been reported in China since 2017. This is due to the “malaria elimination plan 2020” to strengthen surveillance and response, treatment, vector control, personal protection, and health education [28]. A significant challenge to reach total eradication is the import of human malaria cases from the African continent and South-East Asia [29]. Imported dengue fever and chikungunya cases also pose a concern, as the Asian tiger mosquito is established in Beijing, placing these vector-borne diseases in Context 2.

In the Netherlands, native mosquito species such as *Anopheles maculipennis atroparvus* and *Anopheles maculipennis messeae* have the competence of transmitting *Plasmodium vivax*. Despite a peak in imported malaria cases by refugees originating from the horn of Africa in 2015, no autochthonous transmission has occurred. This may be attributed to a low contact rate between infected individuals and competent mosquito species [30].

**Context 3.** Vector present, no pathogen present and no endemic human disease cases

In Beijing and the Netherlands, the presence of a large competent native *Culex pipiens* population places Rift Valley fever in Context 3 for the Netherlands and West Nile fever for China [31,32]. There is theoretical potential for local transmission. Early warning is based upon vector-borne disease diagnostics in clinically suspected animals, such as clusters of dead birds.

**Context 4.** Pathogen present, no vector present and no endemic human disease cases

In Beijing, the vector *Aedes aegypti* is absent, placing yellow fever in Context 4. No field evidence suggests that the Asian tiger mosquito *Aedes albopictus* is a competent vector for this disease. In the Netherlands, Zika, chikungunya and dengue viruses are imported with travellers, but competent vector populations are not established on a wide scale. The invasive exotic *Aedes albopictus* has been introduced into the Netherlands by import mechanisms. These vectors travel in second-hand tires, lucky bamboo plants from China’s tropical southern region, and via the movement of people and goods in international travel. Despite this, *Aedes albopictus* has not become permanently established due to control measures, including the elimination of breeding places and the use of biocides [33,34]. The yellow fever mosquito *Aedes aegypti* is exotic and introduced regularly to the Netherlands, predominantly with planes arriving from native tropical regions, but not invasive to the Netherlands as it requires (sub)tropical climate for establishment [35].

**Context 5.** No endemic human disease cases, with vector and pathogen absent

Both regions have no control measures in place for context 5 vector-borne diseases such as Crimean-Congo haemorrhagic fever. Despite recent findings of individual adult *Hyalomma* ticks on horses, the Netherlands are considered outside the climatic range (the 50° North latitude) for permanent establishment. *Hyalomma scupense*, observed in Beijing, is not competent to transmit the pathogen [36,37].

### Research

In both Beijing and the Netherlands, research is conducted on vectors, pathogens, and human diseases. Environmental and climate data on temperature, rainfall, and humidity are occasionally incorporated to model the expected abundance, activity, occurrence, and potential establishment of vectors.

Beijing’s experts primarily reported ecological and epidemiological research performed on mosquitoes and mosquito-borne diseases respectively. There is less research on ticks and tick-borne diseases, as the latter are not considered a priority in urban Beijing. Examples of research are in the spatial-temporal distribution of vectors, species composition and density, seasonal flights, and transmission capacities of vectors for Japanese encephalitis, dengue and malaria. If research findings show an increased risk for public health, data are used to inform control measures and prioritise future surveillance strategies.

Until 2019, research on ticks and tick-borne diseases, especially Lyme borreliosis and tick-borne encephalitis, had more priority in the Netherlands than ecological and epidemiological research on mosquitoes and mosquito-borne diseases [38]. Ticks and tick-borne diseases are a problem in both rural and urban areas. Mosquito-borne diseases had not been seen as a problem. The upsurge of zoonotic vector-borne diseases such as Usutu and West Nile fever in Europe has recently placed mosquito-borne disease research high on the Dutch research agenda. In the Netherlands, ecological and epidemiological research on mosquito-borne diseases is guided towards a centrally unified approach, consisting of both non-outbreak and outbreak related research.

### Harmonisation and Prioritisation

All experts report that increased harmonisation of research across entomology, public health, and clinical medicine and governmental, academic, national and international research institutions might increase resource efficiency by decreasing overlap and repetition of work. Both regions have a system of harmonised mandatory notification of infectious diseases via internet-based systems. Both focus attention on the disease burden, occurrence of human cases, and outbreak potential, with the threat of emerging diseases often prioritised higher than endemic diseases. Prioritisation is not based on the classification in contexts but the impact and capability of response measures. Therefore, in the Netherlands the prevention of establishment of invasive *Aedes* mosquito populations is a high priority due to the small window of opportunity to achieve and maintain eradication. Control of *Ixodes ricinus*, the latter being competent for transmitting Lyme borreliosis and tick-borne encephalitis, is implemented, only personal protection and education and to lesser degree environmental modification to decrease survival of ticks.

In Beijing, the need for notification is determined according to threat and potential harm to public health driven by the national level, and regional situation [39]. Notifiable vector-borne disease cases are reported via “Notifiable Infectious Diseases Reporting Information System” (NIDRIS) and the “National Vector Surveillance Networks” (NVSN), web-based systems that enable all healthcare and research institutions to report information on notifiable infectious diseases to the public health service [16].

In the Netherlands, the notification procedure is comparable to the Beijing procedure: notifiable vector-borne disease cases are reported via “Online Systeem voor Infectieziekten Registratie binnen ISIS” (OSIRIS). A list of nationally notifiable diseases in China and the Netherlands was published in a study comparing the national infectious disease surveillance systems in the 2 countries [8].

### Response and action

In both regions, response varies with the existence of an outbreak. In an outbreak situation, broad collaboration and increased monetary resources are mobilised. Experts advised that enhanced regional collaboration between institutions and governmental authorities in a non-outbreak situation is desirable as it would improve the preparedness and response to (re)-emerging vector-borne diseases.

Beijing CDC has the responsibility to manage human vector-borne disease cases in Beijing, while the Chinese national CDC has access to vector-borne disease data and can assist if necessary. In a non-outbreak situation, Beijing controls human-biting mosquito species using larvicide (*Bacillus thuringiensis israelensis (B.t.i.)*), as the nuisance caused by their biting is perceived as a public health concern. Vector monitoring occurs three times a month around twenty-two hospitals and control for mosquitoes takes place to reduce nuisance to the public and to reduce public health risk should a vector population threshold be reached. The Beijing government provides funds for its citizens to be vaccinated against Japanese encephalitis as part of the national vaccination scheme. The Chinese government also supports control measures in various African countries to protect overseas Chinese workers from contracting or importing to China such vector-borne diseases as yellow fever. In contrast, this does not occur in the Dutch setting.

In the Netherlands, the regional Public Health Service can report and manage human vector-borne disease cases, except in cases of high-threat notifiable disease or a multi-regional outbreak. In these situations, responsibility shifts to national institutions such as The Netherlands Food and Consumer Product Safety Authority (NVWA) for vector control and the RIVM for disease containment. A special NVWA-task force (known as CMV) monitors invasive *Aedes* mosquitoes in ports, airports, and imported products. A Beijing approach that is not practiced in the Netherlands is the monitoring and control of mosquitos in and around hospitals. While there is no Dutch policy to control native mosquitoes, standing policy to eradicate invasive *Aedes* mosquitoes by removing larval habitats, using adulticides (e.g. *Bacillus thuringiensis israelensis (B.t.i.)* space spray) and larvicides (e.g. *Bacillus sphaericus (B.s.)* granules) has prevented the permanent establishment of the Asian tiger mosquito, *Aedes albopictus* [34]. Government initiatives to vaccinate migrant workers from areas endemic for yellow fever and Japanese encephalitis resemble efforts in Beijing but are not included in the national vaccination scheme. As for Lyme borreliosis, researchers from both regions seek to develop novel treatment and prevention strategies to reduce disease burden.

## Discussion

When focused on vector populations, both Beijing and the Netherlands have a surveillance and response system. The same was identified for human disease cases where surveillance is predominantly centred on the vector-borne diseases transmitted by invasive *Aedes*-mosquitoes. The main focus in the Netherlands is on laboratory confirmed testing once a vector-borne disease case is clinically suspected. In Beijing, syndromic surveillance is used as a gatekeeping mechanism to initially identify and manage cases, later supported by laboratory confirmed diagnosis. Both Beijing and the Netherlands demonstrate preparedness in alignment with the IHR on monitoring at official points of entry such as harbours and airports. Demonstrating additional preparedness for vector-borne diseases by utilising syndromic surveillance is also in alignment with the IHR.

Experts from Beijing’s health system indicate preparedness for imported vector-borne diseases and an awareness of having potentially competent vector species at high-risk locations. This is reflected in actions such as hospital surveillance and public vaccination against Japanese encephalitis. Beijing is prepared for autochthonous vector-borne disease outbreaks, with control measures available for mosquito species.

The Netherlands is comparably prepared for imported vector-borne pathogens, as reflected in the combination of diagnostic tools and awareness towards competent vector species in high-risk locations. Even though the control of established vectors is difficult, the Netherlands informs the public about autochthonous vector-borne diseases whilst stimulating research activities. This effort is reflected in increased public awareness and engagement in citizen science projects such as “Tick-Radar”.

After the interviews were conducted, in 2020 West Nile virus was introduced in the Netherlands and circulated between mosquitoes and birds, with spill over to human resulting in 8 cases [40,41]. In 2021, no indication of West Nile virus circulation was found in intensified surveillance in birds, mosquitoes and humans.

An earlier study on infectious disease surveillance and response found many similarities between China, including Beijing, and the Netherlands [8]. Our findings concur, but there are some differences.

In urban Beijing, tick-borne diseases are not a priority, as the urban environment is not conducive to tick propagation and survival. The increased threat in suburban and rural areas could be attributable to the abundance and diversity of tick species present [42]. New urban planning policies might lead to the introduction of tick populations into urban areas [43]. There is concern that this possibility is overlooked in the planning of urban green spaces in both regions. Beijing’s experts reported a need for increased tick monitoring and risk analysis, while Dutch experts question the feasibility of control, as it is resource-intensive.

Beijing’s experts did not express the need for improved international collaboration, reflecting the more unified and centralised approach within China as opposed to a fragmented approach in Europe. For the Netherlands, it is important to have insight into possible (re)-emerging vector-borne diseases in neighbouring countries, especially in cross-border regions. While there are clear case definitions for vector-borne diseases in the European Union, these are not necessarily adhered to by all EU/EEA Member States [8,44]. In Beijing province, vector-borne disease surveillance is integrated into the national surveillance system (NIDRIS). In contrast, a fragmented vector-borne disease surveillance system is observed in the EU. Since 2019 the ECDC has called for monitoring data to gain more insight into vector-borne diseases incidence and trends in Europe [45].

Vaccines are the most cost-effective preventive control measure available for yellow fever, Japanese encephalitis, and tick-borne encephalitis [46,47]. Free vaccination is assumed to contribute to the low number of human Japanese encephalitis cases in Beijing. The need for generalised tick-borne encephalitis vaccination in Beijing is reported questionable, as ticks thrive only in its rural areas [48]. Dutch guidelines advise a tick-borne encephalitis vaccination for travellers under certain conditions, but not for the Netherlands as cases are still rare. In the general population, vaccination coverage among travellers for this disease is low, perhaps because Dutch citizens perceive a low risk of acquisition outside rural and forest areas of countries like Germany and Austria, where the disease is more common [47,49].

Not all vector-borne diseases have treatments and/or effective vaccines. In both Beijing and the Netherlands, containment of possible epidemics is performed via preventive and reactive strategies. These include the use of biocidal products and use of protective clothing that interrupt the transmission chain or reduce vector density. These region-specific strategies are based on accurate prioritisation of vector-borne diseases in these settings. Knowing the context in which a vector-borne disease occurs in a given area can support tailor-made control measures and inform future preventive strategies. The classification of vector-borne diseases as presented in the framework by Braks et al. and adopted in this paper is conceived as a potentially useful tool to raise awareness and communicate future threats to public health professionals [2].

## Limitations

Three interviews in Beijing were not performed in English, and translation could have led to miscommunication. As 2 researchers conducted interviews, their notes may have contained interpretive differences. This study did not aim to describe vector-borne disease surveillance and response systems in Beijing nor the Netherlands in detail. Instead, it broadly explores the similarities and differences in surveillance and response systems dealing with selected vector-borne diseases and their vectors. This approach could have led to the oversimplification of these systems in both regions. Additionally, we recommend further research comprehensively comparing China with Europe to acknowledge perceived fragmentation in Europe and synergies with the Chinese national system.

We acknowledge that information bias might have occurred as we contacted potential interviewees and asked them if they felt comfortable talking about vectors and/or vector-borne diseases. The interviewees might have provided socially desirable answers.

## Conclusion

The vector-borne disease context framework allowed us to describe and compare the vector-borne disease preparedness between the regions, despite differences in the diseases, pathogens and vectors present. Understanding the vector-borne disease preparedness in different areas can teach us valuable lessons that could support the adaptation and strengthening of national surveillance systems. Although the compared regions deal with different vector-borne disease challenges, the context comparisons made it possible to learn about and enhance knowledge on overseas systems as suggested by the IHR.

## Data accessibility statements

This study did not obtain raw data during the analysis but used data collected in prior studies. This data is not publicly available due to privacy and confidentiality agreements.

## Additional File

The additional file for this article can be found as follows:

10.5334/aogh.3672.s1 Supplement Files. Supplement 1 to 4.

## Abbreviations

**CDC –** Centre for Disease Prevention and Control

**CMV –** Centre for Monitoring Vectors, the Netherlands

**ECDC –** European Centre for Disease Prevention and Control

**EU/EEA –** European Union/European Economic Area

**IHR –** International Health Regulation, WHO

**OSIRIS –** Online Systeem voor Infectieziekten Registratie binnen ISIS

**NVWA –** Netherlands Food and Consumer Product Safety Authority

**RIVM –** National Institute for Public Health and Environment

**VWS –** Ministry of Health, Welfare and Sport, the Netherlands

**WHO –** World Health Organization

## References

[1] The World Health Organization. Vector-borne diseases Factsheets 2017; https://www.who.int/news-room/fact-sheets/detail/vector-borne-diseases. Accessed January, 2019.

[2] Braks M, Medlock JM, Hubalek Z, et al. Vector-borne disease intelligence: strategies to deal with disease burden and threats. Frontiers in Public Health. 2014; 2: 280. DOI: 10.3389/fpubh.2014.00280 25566522 PMC4273637

[3] Fang L-Q, Sun Y, Zhao G-P, et al. Travel-related infections in mainland China, 2014–16: an active surveillance study. The Lancet Public Health. 2018; 3(8): 385–394. DOI: 10.1016/S2468-2667(18)30127-0 PMC7164813 30033200

[4] Rezza G. Dengue and chikungunya: long-distance spread and outbreaks in naive areas. Pathogens and Global Health. 2014; 108(8): 349–355. DOI: 10.1179/2047773214Y.0000000163 25491436 PMC4394667

[5] The World Health Organization. Public Health Surveillance. Health topics 2019. https://www.who.int/topics/public_health_surveillance/en/. Accessed April, 2019.

[6] van Asten L, Fanoy E, Hooiveld M, Koopmans M, Kretzschmar M. Update in Public Health: Syndromic surveillance, a finger on the pulse of public health [in Dutch]. Nederlands Tijdschrift Geneeskunde. 2014; 158: 8. 24975977

[7] Soler M, Fouillet A, Viso A, et al. Assessment of syndromic surveillance in Europe. The Lancet. 2011; 378(9806): 1833–1834. DOI: 10.1016/S0140-6736(11)60834-9 22118433

[8] Vlieg WL, Fanoy EB, van Asten L, et al. Comparing national infectious disease surveillance systems: China and the Netherlands. BMC Public Health. 2017; 17(1): 415. DOI: 10.1186/s12889-017-4319-3 28482830 PMC5423001

[9] May L, Chretien JP, Pavlin JA. Beyond traditional surveillance: applying syndromic surveillance to developing settings–opportunities and challenges. BMC Public Health. 2009; 9: 242. DOI: 10.1186/1471-2458-9-242 19607669 PMC2718884

[10] Fournet F, Jourdain F, Bonnet E, Degroote S, Ridde V. Effective surveillance systems for vector-borne diseases in urban settings and translation of the data into action: a scoping review. Infectious Diseases of Poverty. 2018; 7(1): 99. DOI: 10.1186/s40249-018-0473-9 30217142 PMC6137924

[11] World Data. Netherlands; 2022. https://www.worlddata.info/europe/netherlands/index.php. Accessed June, 2022.

[12] World Data. Climate in Beijing (China); 2022. https://www.worlddata.info/asia/china/climate-beijing.php. Accessed June, 2022.

[13] The People’s Government of Beijing Municipality. Demographic Geography. Facts; 2022. http://english.beijing.gov.cn/beijinginfo/facts/202006/t20200601_1912281.html. Accessed June, 2022.

[14] Braks M, van der Giessen J, Kretzschmar M, et al. Towards an integrated approach in surveillance of vector-borne diseases in Europe. Parasites & Vectors. 2011; 4: 192. DOI: 10.1186/1756-3305-4-192 21967706 PMC3199249

[15] Hartemink N, van Vliet A, Sprong H, et al. Temporal-Spatial Variation in Questing Tick Activity in the Netherlands: The Effect of Climatic and Habitat Factors. Vector Borne Zoonotic Dis. 2019; 19(7): 494–505. DOI: 10.1089/vbz.2018.2369 30810501

[16] Lock-Wah-Hoon J, Zheng Y, Braks M, et al. Exploring Vector-Borne Disease Surveillance and Response Systems in Beijing, China: A Qualitative Study from the Health System Perspective. Int J Environ Res Public Health. 2020; 17(22). DOI: 10.3390/ijerph17228512 PMC7698447 33212908

[17] The World Health Organization. Strengthening health security by implementing the International Health Regulations (2005). Public health at ports, airports and ground crossings; 2019. https://www.who.int/ihr/ports_airports/en/ Accessed April, 2019.

[18] Wu X-B, Na R-H, Wei S-S, Zhu J-S, Peng H-J. Distribution of tick-borne diseases in China. Parasites & Vectors. 2013; 6(119): 8. DOI: 10.1186/1756-3305-6-119 23617899 PMC3640964

[19] Sprong H, Hofhuis A, Gassner F, et al. Circumstantial evidence for an increase in the total number and activity of borrelia-infected ixodes ricinus in the Netherlands. Parasites & Vectors. 2012; 5(294): 11. DOI: 10.1186/1756-3305-5-294 23244453 PMC3562265

[20] Hofhuis A, Bennema S, Harms M, et al. Decrease in tick bite consultations and stabilization of early Lyme borreliosis in the Netherlands in 2014 after 15 years of continuous increase. BMC Public Health. 2016; 16(1): 425. DOI: 10.1186/s12889-016-3105-y 27216719 PMC4877959

[21] National institute for Public Health and Environment (RIVM). Every year, 300 000 tick bites in urban areas; 2017. https://www.rivm.nl/en/news/every-year-300000-tick-bites-in-urban-areas Accessed April, 2019.

[22] Jahfari S, De Vries A, Rijks JM, et al. Tick-Borne Encephalitis Virus in Ticks and Roe Deer, the Netherlands. Emerging Infectious Diseases. 2017; 23(6): 3. DOI: 10.3201/eid2306.161247 PMC5443429 28518024

[23] The World Health Organization. Japanese encephalitis. Fact sheets/Details; 2015. https://www.who.int/news-room/fact-sheets/detail/japanese-encephalitis. Accessed April, 2019.

[24] European Centre for Disease Prevention and Control (ECDC). Facts about Japanese encephalitis Japanese encephalitis; 2019. https://ecdc.europa.eu/en/japanese-encephalitis/facts. Accessed April, 2019.

[25] Rijks JM, Kik ML, Slaterus R, et al. Widespread Usutu virus outbreak in birds in the Netherlands, 2016. Euro Surveill. 2016; 21(45). DOI: 10.2807/1560-7917.ES.2016.21.45.30391 PMC5144937 27918257

[26] Centers for Disease Control and Prevention (CDC). About Malaria. Malaria; 2019. https://www.cdc.gov/malaria/about/faqs.html. Accessed April, 2019.

[27] Feng X, Zhang S, Huang F, et al. Biology, Bionomics and Molecular Biology of Anopheles sinensis Wiedemann 1828 (Diptera: Culicidae), Main Malaria Vector in China. 2017; 8(1473). DOI: 10.3389/fmicb.2017.01473 PMC5552724 28848504

[28] Zhang S, Zhang L, Feng J, et al. Malaria Elimination in the People’s Republic of China: Current Progress, Challenges, and Prospects. In: Towards Malaria Elimination – A Leap Forward. IntechOpen; 2018. DOI: 10.5772/intechopen.77282

[29] Lai S, Sun J, Ruktanonchai NW, et al. Changing epidemiology and challenges of malaria in China towards elimination. Malaria Journal. 2019; 18(1): 107. DOI: 10.1186/s12936-019-2736-8 30922301 PMC6440015

[30] de Gier B, Suryapranata FS, Croughs M, et al. Increase in imported malaria in the Netherlands in asylum seekers and VFR travellers. Malaria Journal. 2017; 16(1): 60. DOI: 10.1186/s12936-017-1711-5 28148300 PMC5288937

[31] Wei X-Q, Liu T, Zhou X-J, Li Q-H, Zhang Y. Investigation of mosquito-vector density and West Nile Virus carrying status in Beijing. Chinese Journal Hygiene Insecticides & Equipments. 2015; 21(6): 5.

[32] Vogels C, Göertz G, Pijlman G, Koenraadt C. Vector competence of European mosquitoes for West Nile virus. Emerging Microbe & Infections. 2017; 6(11): 12. DOI: 10.1038/emi.2017.82 PMC5717085 29116220

[33] Hofhuis A, Reimerink J, Reusken C, et al. The hidden passenger of lucky bamboo: do imported Aedes albopictus mosquitoes cause dengue virus transmission in the Netherlands? Vector Borne and Zoonotic Diseases. 2009; 9(2): 217–220. DOI: 10.1089/vbz.2008.0071 18959501

[34] Scholte EJ, Den Hartog W, Dik M, et al. Introduction and control of three invasive mosquito species in the Netherlands, July-October 2010. Euro Surveillance. 2010; 15(45): 4. DOI: 10.2807/ese.15.45.19710-en 21087591

[35] Ibanez-Justicia A, Gloria-Soria A, den Hartog W, Dik M, Jacobs F, Stroo A. The first detected airline introductions of yellow fever mosquitoes (Aedes aegypti) to Europe, at Schiphol International airport, the Netherlands. Parasites & Vectors. 2017; 10(1): 603. DOI: 10.1186/s13071-017-2555-0 29221490 PMC5723084

[36] The World Health Organization. Introduction to Crimean-Congo Haemorrhagic Fever. In: Formenty DP, (ed.), Managing infectious hazards. Geneva; 2018.

[37] Uiterwijk M, Ibáñez-Justicia A, van de Vossenberg B, et al. Imported Hyalomma ticks in the Netherlands 2018–2020. Parasit Vectors. 2021; 14(1): 244. DOI: 10.1186/s13071-021-04738-x 33962655 PMC8106226

[38] Braks M, van Wieren S, Takken W, Sprong H. Ecology and prevention of Lyme borreliosis. Wageningen: Wageningen Academic Publishers; 2016. DOI: 10.3920/978-90-8686-838-4

[39] The World Health Organization. Infectious diseases. WHO representatives China: Health topics; 2019. http://www.wpro.who.int/china/topics/infectious_diseases/en/. Accessed April, 2019.

[40] Vlaskamp DR, Thijsen SF, Reimerink J, et al. First autochthonous human West Nile virus infections in the Netherlands, July to August 2020. Eurosurveillance. 2020; 25(46): 2001904. DOI: 10.2807/1560-7917.ES.2020.25.46.2001904 PMC7678035 33213687

[41] Sikkema RS, Schrama M, van den Berg T, et al. Detection of West Nile virus in a common whitethroat (Curruca communis) and Culex mosquitoes in the Netherlands, 2020. Euro surveillance: bulletin Europeen sur les maladies transmissibles = European communicable disease bulletin. 2020; 25(40). DOI: 10.2807/1560-7917.ES.2020.25.40.2001704 PMC7545818 33034280

[42] Zhao J, Wang H, Wang Y. Regional distribution profiles of tick-borne pathogens in China. Chinese Journal of Vector Biology & Control. 2012; 5: 3.

[43] Gao P, Pilot E, Rehbock C, et al. Land use and land cover change and its impacts on dengue dynamics in China: A systematic review. PLOS Neglected Tropical Diseases. 2021; 15(10): e0009879. DOI: 10.1371/journal.pntd.0009879 34669704 PMC8559955

[44] The European Commission. COMMISSION IMPLEMENTING DECISION (EU) 2018/945 of 22 June 2018 on the communicable diseases and related special health issues to be covered by epidemiological surveillance as well as relevant case definitions. In: Commission TE (ed.), Brussels 2018.

[45] European Centre for Disease Prevention and Control (ECDC). ECDC comment: European Commission updates communicable disease surveillance list – Lyme neuroborreliosis now under EU/EEA surveillance. News & events; 2018. https://ecdc.europa.eu/en/news-events/ecdc-comment-european-commission-updates-communicable-disease-surveillance-list-lyme. Accessed April, 2019.

[46] Aggarwal A, Garg N. Newer Vaccines against Mosquito-borne Diseases. The Indian Journal of Pediatrics. 2018; 85(2): 117–123. DOI: 10.1007/s12098-017-2383-4 28560654

[47] The World Health Organization. Vaccines against tick-borne encephalitis: WHO position paper. The World Health Organization; 2011.

[48] Xing Y, Schmitt HJ, Arguedas A, Yang J. Tick-borne encephalitis in China: A review of epidemiology and vaccines. Vaccine. 2017; 35(9): 1227–1237. DOI: 10.1016/j.vaccine.2017.01.015 28153343

[49] National institute for Public Health and Environment (RIVM). Tekenencefalitis Richtlijn. Richtlijnen & Draaiboeken; 2019. https://lci.rivm.nl/richtlijnen/tekenencefalitis. Accessed April, 2019.

