# Supplementary material for: Comparing Vector-Borne Disease Surveillance and Response in Beijing and the Netherlands
Source: Ann Glob Health. 2022 Jul 26;88(1):59. doi: 10.5334/aogh.3672 (PMC9336689; doi:10.5334/aogh.3672)
Supplement: Supplement Files. — Supplement 1 to 4. [file agh-88-1-3672-s1.pdf]

Supplement 1

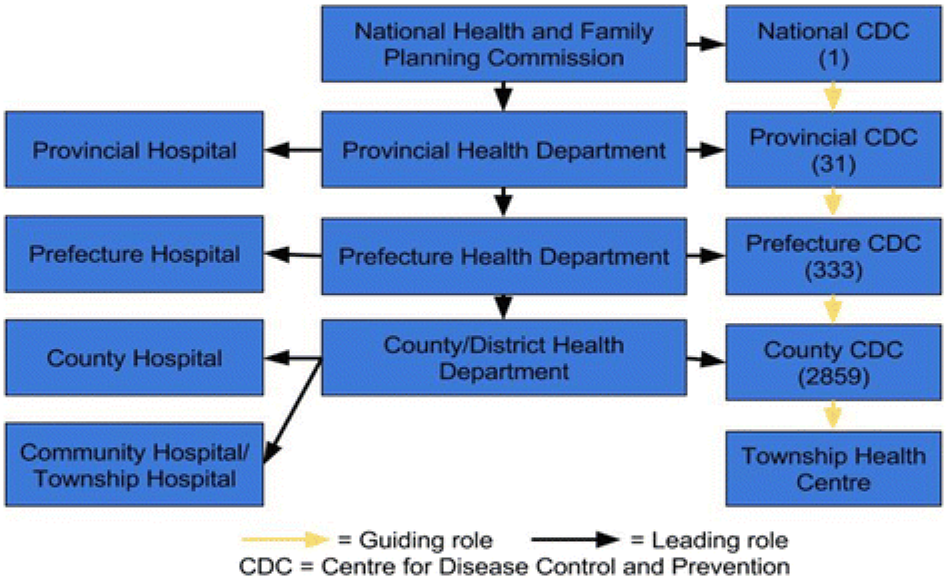

Figure 1. The organisation of Chinese public health organisations involved in infectious disease control (figure reproduced from Vlieg et al., 2017)[8]

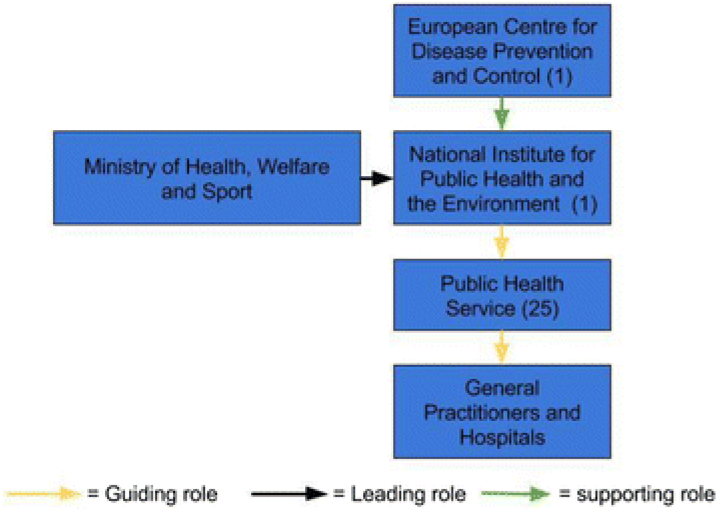

Figure 2. The organisation of Dutch public health organisations involved in infectious disease control (figure reproduced from Vlieg et al., 2017)[8]

Supplement 2

Expert	Profession	Institution	Focus of interview	Governance level	Region
1	Parasitologist	Beijing CDC	Malaria (*)	Province	Beijing
2	Clinician	Ditan Hospital	Primary care	Province	Beijing
3	Epidemiologist	Chaoyang CDC	Vector-borne diseases	District	Beijing
4	Epidemiologist	Beijing CDC	Lyme disease	Province	Beijing
5	Professor	Shun Yi CDC	Vector-borne diseases (*)	District	Beijing
6	Immunologist	Beijing CDC	Japanese Encephalitis (*)	Province	Beijing
7	Assistant professor	China National CDC	Vector-borne diseases	National	China
8	Epidemiologist	Beijing CDC	Vectors	Province	Beijing
9	Epidemiologist	Beijing CDC	Surveillance systems	Province	Beijing
10	Laboratory scientist	Beijing CDC	Vector-borne diseases	Province	Beijing
11	MD/epidemiologist	RIVM/GGD	Lyme disease	National	NL
12	Epidemiologist	RIVM	Lyme disease	National	NL
13	Medical doctor	RIVM	Zika	National	NL
14	Biologist	RIVM	Lyme disease	National	NL
15	Epidemiologist	RIVM	Zika	National	NL
16	Biologist	CMV-NVWA	Vectors	National	NL
17	Biologist	Self-employed	Lyme disease	National	NL
18	Biologist	Erasmus MC	West-Nile virus	National	NL
19	Policy officer	Municipality	Vectors	Regional	NL

Table 1. Characteristics of interviewed experts from Beijing, China and the Netherlands in the first half of 2018. (*) Indicates the use of a translator during the interviews

	Key-terms
Language	Publication language: ‘English’, ‘Dutch’
Year	Publication year between ‘2009 – 2019’
Countries/States/Continents	‘Europe’, ‘The Netherlands’, ‘China’, ‘Beijing’

Inclusion	‘Vectors’, ‘Vector-borne diseases’, ‘Mosquitos’, ‘Mosquito-borne diseases’, ‘Ticks’, ‘Tick-borne diseases’, ‘Public Health surveillance’, ‘Infectious diseases surveillance’, ‘Vector-borne surveillance’, ‘Vector control’, ‘Early prevention’, ‘Global trade and travel’, ‘Climate and environmental change’, ‘Response systems’ and ‘Predictive surveillance’.
Exclusion	‘Blackfly-borne diseases’, ‘Sandfly-borne diseases’, ‘Triatomine-borne diseases’, ‘Tsetse fly-borne diseases’, ‘Water snail-borne diseases’, ‘Rodent-borne diseases’ and ‘Various and undefined-borne diseases’.

Supplement 3

Table 2. Key-terms used for literature review to obtain supplementary information

Supplement 4

Category	Autochthonous human cases	Pathogen occurrence (in local reservoir animals or frequent import of infectious persons or animals)	Vector present
1 a. Endemic<br>1 b. Epidemic/anecdotal	+	+	+
2	-	+	+
3	-	-	+
4	-	+	-
5	-	-	-

Table 3. Vector-borne disease context matrix on the current presence (√) or absence (-) of endemic (human) disease, pathogen and vector (Braks et al., 2011)[14]

1
